# Are we still here?

**DOI:** 10.4103/0972-124X.70825

**Published:** 2010

**Authors:** D. Arunachalam

**Affiliations:** *Editor, Journal of Indian Society of Periodontology, H 11 A South Avenue, Thiruvanmiyur, Chennai - 600 041, India. E-mail: editor@jisponline.com*

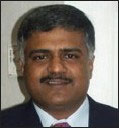



In an article in General Dentistry in May 2010, Dr. Louis Malcmacher lamented, “Where did all the periodontists go?”[1]

Dr. Malcmacher is a renowned speaker on various aspects of general dentistry. He is in continuous communication with general dentists and specialists across the US and had a genuine reason to be concerned. Among the many periodontists who he had spoken to during his lectures over the last two years, a majority had said that ‘they would rather remove teeth and place implants than actually treat patients through traditional periodontal surgery and try having them maintain their dentition’.

## DOES IT EVOKE A STRONG EMOTIONAL REACTION? I BET IT DOES!

Does the paradigm of the implant possibility deter classical periodontists from their aim of preserving the natural dentition? Is the periodontist the patient’s partner in retaining the existing oral health or has he a set design for an alternative dentition? Are we divided among those that proffer implants through ‘evidence base’ of 94% success rates and those that decry implant use, citing alternative ‘evidence bases’?

These are the real thought-provoking questions that need just that – thought.

Looking back – the theory of exquisite plaque control and meticulous mechanical debridement has been turned into a ‘myth’ by the recent epigenetic theory squarely naming inflammation as the primary modifier of periodontal disease. What is more? This has led to linking the perio-systemic conundrum to inflammation.

## WHAT HAVE WE DONE IN PERIODONTICS TO SAVE TEETH?

How about aesthetic periodontal surgery, functional preprosthetic surgery, osseous grafting, guided bone and tissue regeneration, mucogingival procedures to cover denuded roots, growth factors and biologics, systemic antibacterial therapy, local drug delivery, advanced instrumentation, and the list goes on.

## HOW HAVE WE CONTRIBUTED TO IMPLANT DENTISTRY?

Well, substantially, considering the advances in aesthetic positioning of implants, soft and hard tissue surgery of implants, grafting procedures, implant maintenance, and management of failing implants.

The golden mean then seems to be the ability to utilise what we have learnt from periodontal regeneration and disease management and incorporate it into implant dentistry to complement, and not replace, periodontal therapy[2]—a sentiment echoed by President of the AAP, Sam Low.

There is another sentiment, and a well-deserved one, where, general dentists believe that it is as important to assess the personality of patients and determine their orientation to oral health and the stakes they are willing to take in keeping their overall health. Dr. Malcmacher believes that is far more important than all the scientific evidence, periodontal condition, and quality or quantity of bone. That he claims ‘is somewhat left out of the conversation’.

Strangely, but truly, he may be more than just right. We hope that we have not gone so close to the trees, that we have lost sight of the forest.
